# Combined Molecular and Elemental Mass Spectrometry Approaches for Absolute Quantification of Proteomes: Application to the Venomics Characterization of the Two Species of Desert Black Cobras, *Walterinnesia aegyptia* and *Walterinnesia morgani*

**DOI:** 10.1021/acs.jproteome.1c00608

**Published:** 2021-10-04

**Authors:** Juan J. Calvete, Davinia Pla, Johannes Els, Salvador Carranza, Maik Damm, Benjamin-Florian Hempel, Elisa B. O. John, Daniel Petras, Paul Heiss, Ayse Nalbantsoy, Bayram Göçmen, Roderich D. Süssmuth, Francisco Calderón-Celis, Alicia Jiménez Nosti, Jorge Ruiz Encinar

**Affiliations:** † Laboratorio de Venómica Evolutiva y Traslational, Instituto de Biomedicina de Valencia, Consejo Superior de Investigaciones Científicas (CSIC), Jaume Roig 11, 46010 Valencia, Spain; ‡ Environment and Protected Areas Authority, 82828 Sharjah, United Arab Emirates; § Institute of Evolutionary Biology, CSIC-Universitat Pompeu Fabra, 08003 Barcelona, Spain; ∥ Department of Chemistry, Technische Universität Berlin, 10623 Berlin, Germany; ⊥ BIH Center for Regenerative Therapies BCRT, Charité-Universitätsmedizin Berlin, 13353 Berlin, Germany; # Center of Biotechnology, Universidade Federal do Rio Grande do Sul, CEP 91501-970 Porto Alegre, RS, Brazil; ∇ CMFI Cluster of Excellence, Interfaculty Institute of Microbiology and Medicine, University of Túbingen, 72076 Tübingen, Germany; ○ Department of Bioengineering, Faculty of Engineering, Ege University, 35100 Bornova, Izmir, Turkey; ◆ Zoology Section, Department of Biology, Faculty of Science, Ege University, 35100 Bornova, Izmir, Turkey; ¶ Department of Physical and Analytical Chemistry, University of Oviedo, 33006 Oviedo, Spain

**Keywords:** snake venomics, combined top-down and bottom-up venomics, hybrid elemental and molecular mass spectrometry, absolute quantification of venom proteome, desert black cobra, Walterinnesia aegyptia, Walterinnesia morgani

## Abstract

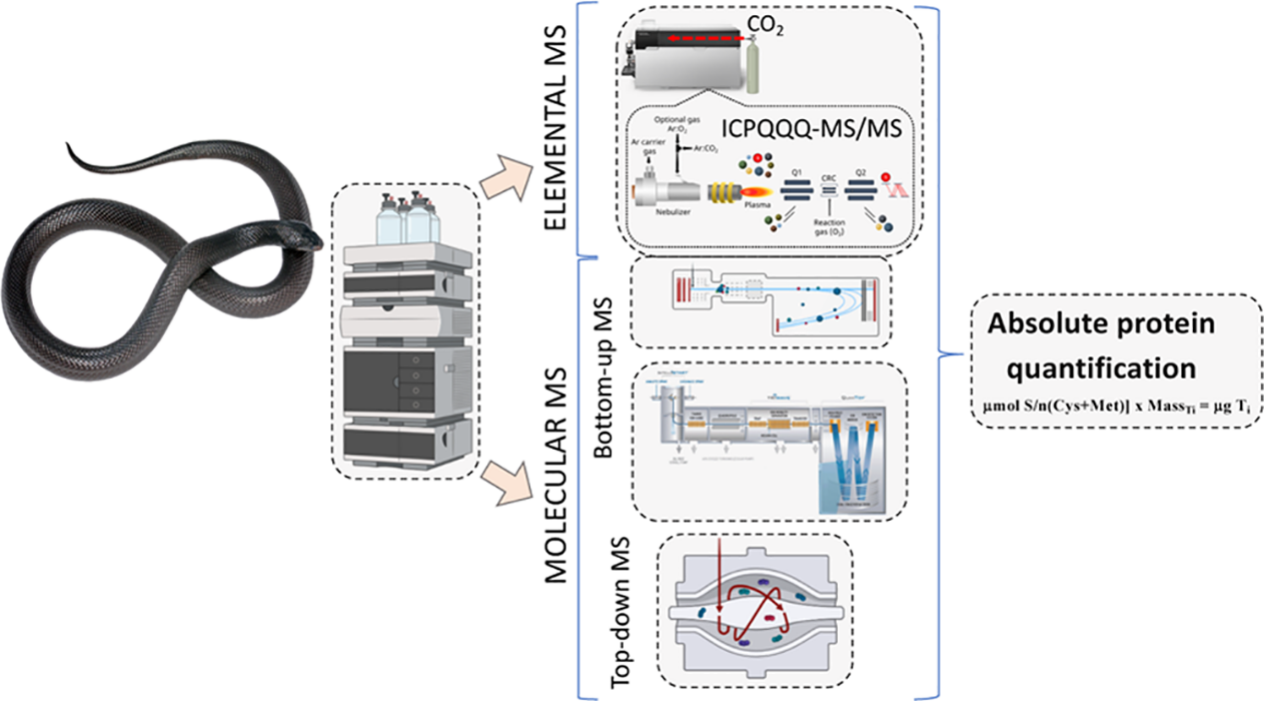

We report a novel hybrid, molecular and elemental mass spectrometry (MS) setup for the absolute quantification of snake venom proteomes shown here for two desert black cobra species within the genus *Walterinnesia*, *Walterinnesia aegyptia* and *Walterinnesia morgani*. The experimental design includes the decomplexation of the venom samples by reverse-phase chromatography independently coupled to four mass spectrometry systems: the combined bottom-up and top-down molecular MS for protein identification and a parallel reverse-phase microbore high-performance liquid chromatograph (RP-μHPLC) on-line to inductively coupled plasma (ICP-MS/MS) elemental mass spectrometry and electrospray ionization quadrupole time-of-flight mass spectrometry (ESI-QToF MS). This allows to continuously record the absolute sulfur concentration throughout the chromatogram and assign it to the parent venom proteins separated in the RP-μHPLC-ESI-QToF parallel run via mass profiling. The results provide a locus-resolved and quantitative insight into the three desert black cobra venom proteome samples. They also validate the units of measure of our snake venomics strategy for the relative quantification of snake venom proteomes as % of total venom peptide bonds as a proxy for the % by weight of the venom toxins/toxin families. In a more general context, our work may pave the way for broader applications of hybrid elemental/molecular MS setups in diverse areas of proteomics.

## 1 Biological Significance

The development of quantitative protocols during the first decade of the 21st century has represented a major advance in the field of proteomics. However, given the inherently nonquantitative nature of molecular mass spectrometry (MS), the absolute quantification of the proteome components is still a challenge. Absolute quantification through molecular MS requires spiking the experimental sample with a certified concentration of an isotopically labeled version for each target molecule. To address the mismatch between the limited quantification capabilities of molecular MS platforms and the requirements of modern venomics, we have incorporated inductively coupled plasma (ICP)-MS, a well-known technique in the field of bioinorganic elemental analysis, into a novel hybrid, molecular and elemental liquid chromatography–mass spectrometry (LC–MS) workflow. This combines the unparalleled molecular resolution of top-down MS and the absolute quantification of sulfur (S) by ICP-MS as a proxy for the absolute quantification of venom proteomes using a generic sulfur standard. As a proof-of-concept, we have applied this novel strategy to quantify three venom proteomes of the two desert black cobra species within the genus *Walterinnesia*.

## 2 Introduction

Venoms and their associated venom-delivery systems are intrinsically ecological traits that have evolved independently in a wide range of lineages across all major phyla of the animal tree of life.^1,2^ Every ecosystem of our planet where there is competition for resources harbors animals possessing toxic weaponry. Venoms should therefore be understood as adaptive responses shaped by natural selection in the context of reciprocal selective predator–prey pressures that maximize the venomous organisms’ fitness in local environments through optimization of the foraging/risk-of-predation balance and/or the self-defense from predators.^3−9^

Sixty to 50 million years ago, in the wake of the Cretaceous–Paleogene boundary mass extinction event that ended the reign of nonavian dinosaurs,^10,11^ the emergence of venom represented a key evolutionary innovation underpinning caenophidian snake radiation.^12,13^ Extant snake venoms are integrated phenotypes comprised of mixtures of dozens to hundreds of peptides and proteins, collectively referred to as toxins, which, despite belonging to a limited number (2 < *n* < 20) of protein families, possess a wide range of potent and specific pharmacological activities capable to wreak havoc on the vital systems of the animal prey or human victim.^14−18^

Snakebite envenoming is an occupational hazard and a disease of poverty that annually claims over 100 000 human lives worldwide, particularly in the tropical and subtropical African and Asian rural regions where ecological interactions between venomous snakes and local people engaged in rural activities are frequent.^19−22^ Snakebite envenoming represents a multifactorial One Health challenge.^20^ Integration and contextualization of conceptual frameworks from ecological venomics and clinical toxinology can be mutually enlightening if snakebite envenoming is analyzed from an ecological stance.^23^ Hence, identifying the specific pressures that tailored the composition and bioactivities of venoms across snake clades may have implications for the clinical treatment of human envenomings.^24−27^

Abundance and toxicity are conjugated parameters of the reference frame that explain the individual or synergistic pharmacological profile of venom toxins. Since the turn of the 21st century, knowledge gathered from applications of omics technologies, particularly the combination of next-generation transcriptomics and mass spectrometry (MS)-based proteomics platforms, has yielded compositional insights into snake venoms from 238+ nominal species, mostly within the families Viperidae (340 species of true vipers and pit vipers) and Elapidae (360 species of cobras, kraits, mambas, and sea snakes).^23,28,29^ However, in contrast to the unprecedented highly resolved descriptive knowledge of the compositional diversity of venoms, estimating the abundance of their individual toxins has not followed a parallel advance, e.g., toward absolute quantification.^30−32^ This is because MS is not an inherently quantitative technique. A number of confounding factors may contribute to the quantification of peptide ions in a mass spectrometer. Hence, different analytes in any given sample may have different and unpredictable ionization potentials, and the detection efficiencies for different *m*/*z* signals are unequal.^33,34^ Absolute quantification through molecular MS requires, for each target biomolecule, spiking the experimental sample with a corresponding “Protein Standard for Absolute Quantification” (PSAQ). PSAQs are whole synthetic isotopically labeled analogues of the proteins to be quantified of certified concentration and similar ionization efficiency as the target analyte.^35,36^ The recombinant or synthetic production of PSAQs for each of the proteoforms of a venom proteome may be technically possible, but it is not a feasible option in practice.

To address the mismatch between the quantification capabilities of molecular MS-based proteomics platforms and the requirements of modern venomics applications, we have incorporated inductively coupled plasma (ICP)-MS, a well-known technology in the field of bioinorganic elemental analysis, into hybrid molecular and elemental LC–MS workflows for the determination of sulfur (S) via isotope dilution analysis (IDA)^37−39^ as a proxy for the absolute quantification of venom proteomes using a generic sulfur standard.^40,41^ From the absolute quantification of sulfur, the absolute amount of the parental biomolecule can be calculated if the molar ratios of the S-containing amino acids (cysteine and methionine) are known. In this work, we have applied a recently developed strategy where IDA has been replaced by the addition of C-containing gas mixture (Ar/CO_2_) directly to the plasma to compensate for changes in the organic composition of the mobile phase along the reverse-phase (RP) chromatographic acetonitrile (ACN) gradient.^42,43^ This novel instrumental setup provides a stable and corrected chromatographic signal, which is a simpler and more easily automatable configuration than IDA and has enhanced sensitivity compared to previous strategies. In this work, we have applied this novel strategy to quantify the venom proteomes of the two species of genus *Walterinnesia*.

The western species, *Walterinnesia aegyptia* Lataste 1887,^44^ is found in rocky and mountainous deserts, gravel and sandy plains, and vegetated wadis in Egypt, border areas of Syria, Jordan, Israel, and Palestine, while the eastern species, *Walterinnesia morgani* (Mocquard, 1905), ranges from Syria, Turkey, and northern Iraq, to Iran.^45,46^ The situation in Saudi Arabia is somewhat more confusing. Although Nilson and Rastegar-Pouyani^46^ draw a line separating the ranges of both species based on some morphological characters, the fact that all Saudi Arabian juveniles of *Walterinnesia* are black without the typical narrow pinkish-brown cross-bands that characterize the juvenile specimens of *W. morgani* from outside Saudi Arabia (including the type locality in western Iran)^47^ challenges Nilson and Rastegar-Pouyani’s 2007 hypothesis^46^ and suggests that all Saudi Arabian specimens might belong to *W. aegyptia*.^48,49^

Desert black snakes are medium-sized (maximum size of 1.3 m, average 0.8–1.2 m) strictly terrestrial snakes characterized by a largely nocturnal and fossorial mode of life.^49^ They are quick-moving snakes that actively prey at night on dhub or spiny-tailed lizards, toads, snakes, and occasionally birds and mice.^44,50^ Desert black snakes usually bite their prey sideways at short distances and often use constriction in addition to their potent (intraperitoneally, i.p. LD_50_ of 0.175 mg/kg in 200–250 g adult male albino rats) neurotoxic venom to kill the prey.^51^ Desert black snakes are reluctant to strike but will bite if cornered or threatened. A few bite cases, although no recent fatalities, have been documented. Bites may result in localized pain and swelling, fever, generalized weakness, respiratory distress, double vision, nausea, and vomiting.^50,52,53^ The comprehensible venomics characterization here reported may lay the groundwork for future toxicovenomics analysis that defines the functional map of the venoms of the black desert snakes.

## 3 Materials and Methods

### 3.1 Venoms and Reagents

Venom samples of Saudi Arabian *W. aegyptia* [CN6136 (adult female, Riyadh)], Egyptian Sinai Peninsula [CN6137 (adult male), CN6138 (adult female), CN6139 (juvenile male), and CN6140 (juvenile female)] were obtained, with permission and under the supervision of the Environment and Protected Areas Authority, Government of Sharjah (UAE), from the live collection maintained at the Breeding Centre for Endangered Arabian Wildlife. To clarify the taxonomy of the specimen from Riyadh, Saudi Arabia, two mitochondrial (16S rRNA and cytochrome oxidase I) and one nuclear (melanocortin 1 receptor) genes were PCR amplified and sequenced for specimen CN6136 from Riyadh and specimen CN6137 from the Sinai (see above) using the same primers and conditions.^54,55^ The sequences of the two specimens were nearly identical, presenting one change in 530 base pairs (bp) of the 16S rRNA, one change in 669 bp of the cytochrome oxidase I; and 0 changes in 686 bp of the melanocortin 1 receptor (GenBank Accession numbers MZ520318–MZ520323). The results of the comparison of the mitochondrial and nuclear DNA data unambiguously identify the snake sample from Riyadh as *W. aegyptia* and clearly show that the taxonomic hypothesis of the genus *Walterinnesia* and especially the division between *W. aegyptia* and *W. morgani* within Saudi Arabia by Nilson and Rastegar-Pouyani^46^ is incorrect and should be revised using molecular data.

The venom of *W. morgani* was collected from one adult female captured in November 2007 near Çörten village at Kilis and Gaziantep province boundaries^56^ and maintained since in captivity at the Reptile Biology and Ecology Research Laboratory (Zoology Section, Department of Biology, Ege University). Venom was obtained by allowing the snakes to bite a paraffin-covered laboratory beaker without pressing the venom glands. The venom sample was centrifuged at 4 °C at 2000*g* for 10 min, and the supernatants were immediately lyophilized and the samples stored at 4 °C. The Ege University Local Ethics Committee (process number 2013-050) approved the experimental protocol.

Inorganic sulfur ICP standard (1000 mg/L) was purchased from SPEX CertiPrep, INC. (New Jersey). Solutions were prepared in ultrapure (18.2 MΩ·cm) water. HPLC grade acetonitrile (ACN) was purchased from Fischer Scientific, and formic acid (FA) was purchased from Merck KGaA (Germany).

### 3.2 Determination of the Murine Median Lethal Dose (LD_50_) of *W. morgani* Venom

The murine median lethal dose (LD_50_) of pooled *W. morgani* crude venom was determined through the up-and-down method recommended by the Organization for Economic Cooperation and Development (OECD) Guidelines (Test No. 425).^57,58^ To this end, increasing venom amounts (0.1, 1, and 5 mg of total venom proteins per kg of mouse body weight) dissolved in 100 μL of physiological (0.9%) saline solution were administered intraperitoneally (i.p.) to groups of five Balb/c mice. Control mice received a single i.p. injection of sterile saline (0.9%, 100 μL). Deaths were recorded 24 h after venom injection, and the LD_50_ was calculated through a nonlinear regression fitting procedure in GraphPad Prism 5 (version 5.01).

### 3.3 Molecular Mass Spectrometric Characterization of the Venom Arsenals of the Desert Black Snakes, *W. aegyptia* and *W. morgani*

Initial reverse-phase chromatographic profiling of the five *W. aegyptia* venom samples showed a conserved protein elution pattern in the four Egyptian Sinai Peninsula specimens [CN6137 (adult male), CN6138 (adult female), CN6139 (juvenile male), and CN6140 (juvenile female)], and a different pattern for the venom of the adult female specimen from Riyadh (CN6136). Venoms of this Saudi Arabian snake and the Egyptian adult male specimen CN6137 were selected for comparing their proteome toxin composition between themselves and with the venom proteome of the adult female *W. morgani* (Çörten village, Turkey) specimen.

#### 3.3.1 Bottom-Up Decomplexation and Relative Quantification of the *W. aegyptia* and *W. morgani* Venom Proteomes

For reverse-phase chromatographic decomplexation, 2 mg of crude lyophilized venom samples was dissolved in 100 μL of 0.05% trifluoroacetic acid (TFA) and 5% acetonitrile, and the insoluble material was spun down in an Eppendorf centrifuge at 13 000*g* for 10 min at room temperature. Decomplexation of the venom proteomes was performed according to the reverse-phase high-performance liquid chromatography (RP-HPLC)/sodium dodecyl sulfate-polyacrylamide gel electrophoresis (SDS-PAGE) protocol of our “snake venomics” strategy^59,60^ with minor modifications.^61^ To this end, 40 μL was applied to a RP-HPLC Teknokroma Europa C18 (250 mm × 4 mm, 5 μm particle size, 300 Å pore size) column. The venom proteins were fractionated using an Agilent LC 1100 high-pressure gradient chromatography system equipped with a diode array detector, applying a linear gradient of 0.1% (v/v) TFA in water (solution A) and in 70% acetonitrile (solution B): 0–5 min isocratically with 5% B (0.1% TFA in ACN), followed by the following linear gradient steps: 5–25% B (10 min), 25–45% B (60 min), and 45–70% (10 min), at 1 mL/min. Protein peaks were recorded at λ = 215 nm, and the eluate was manually collected and dried using a vacuum centrifuge (SpeedVac, Thermo Savant).

Molecular masses of the RP-HPLC-separated venom proteins were estimated by nonreducing and reducing SDS-PAGE (on 15% polyacrylamide gels) or determined by nano-Acquity UltraPerformance LC (UPLC) equipped with a BEH130 C_18_ (100 μm × 100 mm, 1.7 μm particle size) column in-line with a Waters SYNAPT G2 high-definition mass spectrometer, as previously described.^62^

Protein bands of interest were excised from Coomassie Brilliant Blue-stained SDS-PAGE gels and subjected to automated in-gel reduction and alkylation using a Genomics Solution ProGest Protein Digestion Workstation.^62^ Tryptic digests were submitted to MS/MS analysis using the same Mass Spectrometry System and chromatographic separation conditions as above. Doubly and triply charged ions were selected for CID-MS/MS. Fragmentation spectra were submitted to the MASCOT Server (version 2.6) at http://www.matrixscience.com and matched against the last update of the NCBI nonredundant database, including the *W. aegyptia* venom gland transcriptomic data deposited with the SRA and TSA databases of NCBI (BioProject accession number PRJA506018) publicly available in the MassIVE repository under the accession number MSV000081885 (ftp://massive.ucsd.edu/MSV000081885/) and ProteomeXchange with the accession number PXD008597. Good quality unmatched fragmentation spectra were manually (*de novo*) sequenced, and the assigned peptide sequences matched to homologous snake venom proteins available in the NCBI nonredundant protein sequences database using the default parameters of the BLASTP program (https://blast.ncbi.nlm.nih.gov/Blast.cgi).^62^

For the relative quantification of the venom arsenals of *W. aegyptia* and *W. morgani*, we applied the three-step hierarchical venom proteome quantification protocol developed in our laboratory^60,63^ to compile the relative composition of toxin families in the venom proteome of *W. aegyptia* and *W. morgani* venom samples. The calculated relative abundances correspond to the % by weight (g/100 g) of the pure venom component.^41^

#### 3.3.2 Top-Down Venomic (TDV) Analysis of the *W. aegyptia* and *W. morgani* Venom Weaponry

Denaturing top-down proteomic experiments were performed as previously described.^58,64−66^ In short, 100 μg of crude venoms was dissolved at a final concentration of 10 mg/mL in aqueous 1% (v/v) formic acid (FA). Dissolved venom was centrifuged at 20 000*g* for 5 min, and the supernatant was mixed with 30 μL of citrate buffer (0.1 M, pH 3.0). For reduction of disulfide bonds, 10 μL of 0.5 M tris(2-carboxyethyl)phosphine (TCEP) was added to one-half of the sample and incubated for 30 min at 65 °C. The other half of the sample was supplemented with 10 μL of ultrapure water. The samples were centrifuged at 20 000*g* for 5 min, and 10 μL of reduced and nonreduced samples was analyzed each by LC (RP-HPLC)-high-resolution (HR) electrospray ionization (ESI)-MS/MS. Technical duplicates were performed by two different LC-ESI-HR-MS setups, *W. aegyptia* venoms by setup (A) and *W. morgani* venom by setup (B).

Setup (A) was performed in an LTQ Orbitrap XL mass spectrometer (Thermo, Bremen, Germany) coupled to an Agilent 1200 HPLC system equipped with a Supelco Discovery 300 Å C18 (2.1 mm × 150 mm, particle size, 3 mm) column. The column was developed with a gradient of 0.1% FA in water (solution A) and acetonitrile (ACN) (solution B) at a flow rate of 0.3 mL/min. Chromatographic conditions and ESI settings were as previously described.^65^

Setup (B) LC–MS/MS experiments were done using a Vanquish ultra-high-performance liquid chromatography (UHPLC) system equipped with a 300 Å pore size, 2 mm × 150 mm column size, 3 μm particle size Supelco Discovery BIO wide C18 column thermostatted at 30 °C and hyphenated to a Q-Exactive quadrupole orbital ion trap (Thermo Fisher Scientific) as previously described.^66^ MS/MS spectra were obtained in the DDA mode at a mass resolution of 140 000 (at *m*/*z* 200), and the three most abundant ions of the survey scan were selected for MS/MS.

##### 3.3.2.1 Top-Down MS Analysis and Intact Mass Profiling

Thermo data (.raw) were converted to a centroided mass spectrometry data format (.mzXML) using the MSconvert software of the ProteoWizard package (http://proteowizard.sourceforge.net; version 3.0.10577)^67^ with a peak picking level of 1+. The. mzXML data were deconvoluted to a. msalign file using TopFD (http://proteomics.informatics.iupui.edu/software/toppic/; version 1.3) with a maximum charge of 50, a maximum mass of 100 000 Da, an MS1 S/N ratio of 3.0, an MS2 S/N ratio of 1.0, an *m*/*z* precursor window of 3.0, and an *m*/*z* error of 0.02. The final sequence annotation was performed with TopPIC (http://proteomics.informatics.iupui.edu/software/toppic/; version 1.3),^68^ with decoy database, 15 ppm mass error tolerance, *E*-value cutoff at 0.01 by E-value computation, 1.2 Da PrSM cluster error tolerance, and a maximum of 2 mass shifts (±500 Da). Spectra were matched against a *W. aegyptia* database as well as against a reviewed Elapinae database (https://www.uniprot.org/, 518 entries, 20.12.2020), manually validated, and visualized using the MS and MS/MS spectra using Qual Browser (Thermo Xcalibur 2.2 SP1.48) and Freestyle (Thermo Xcalibur 1.6.75.20). The XTRACT algorithm of Thermo Xcalibur was used to deconvolute isotopically resolved spectra.

### 3.4 Absolute Quantification of Sulfur by Capillary RP-HPLC On-Line to Inductively Coupled Plasma (ICP-MS/MS) Elemental Mass Spectrometry

Lyophilized venom samples were reconstituted in ultrapure water to a final sample concentration of ∼0.5 mg/mL. The venom proteins contained in 1 μL were separated by RP-HPLC using a Sigma-Aldrich (Steinheim, Germany) 150 mm × 0.3 mm C4 capHPLC column (BIOShell A400, 3.4 μm particle size, 400 Å pore size) run on an Agilent Technologies (Waldbronn, Germany) Infinite Capillary HPLC 1260 Series system equipped with an autosampler module and a Spark Holland oven heating system (Mistral, the Netherlands). The column was developed at 80 °C at a flow rate of 4.5 mL/min with a gradient of 0.2% FA in water (solution A) and 0.2% FA in acetonitrile (solution B). Optimized chromatographic conditions (min % B) were as follows: *W. aegyptia* [CN6137 (adult male, Sinai Peninsula, Egypt)] 0–2, 2–2, 4–8, 11–15, 13–16, 17–16, 27–18, 39–22, 57–35, 67–60, and 73–90; *W. aegyptia* [CN6136 (adult female, Riyadh, Saudi Arabia)] 0–1.5, 5–2, 6–10.8, 13–11.1, 15–24.1, 27–24.2, 28–29.2, 35–29.5, 38–65, 43–75, and 45–90; *W. morgani* (adult female, Çörten village, Turkey) 0–1.5, 5–1.5, 8–10, 18–15, 30–25, 45–30, 55–70, and 60–90. Complete protein recovery from the chromatographic column, an essential requisite to accurately quantify venom proteins with ICP-MS, was assessed by injecting in triplicate the sample under flow injection analysis (FIA) prior to the chromatographic analysis.^40^ Capillary RP-HPLC FIA conditions were the same as the starting conditions for venom decomplexation, and both RP-HPLC fractionation and FIA analysis used the same sample injection volume so that sulfur mass balance could be directly determined.

For the absolute quantification of the venom components, sulfur was continuously quantified through ICP-MS/MS analysis.^69^ The analytical potential of sulfur measurement for the general quantitative analysis of cysteine and/or methionine-containing proteins and peptides^70^ has already been validated for venom proteome quantification.^40^ The ICP-MS/MS system used was an Agilent 8900 triple quadrupole ICPQQQ-MS (Tokyo, Japan). The sulfur quantification standard was injected using capFIA prior to the capHPLC analysis.^43^ This standard can be any compound of certified concentration that contains sulfur because of the species-independency of the elemental response in the detection. External calibration provided the sulfur response factor (i.e., the peak area of S per unit of concentration of the S standard injected) and was applied using eq 1 to quantify the sulfur present in each chromatographic peak of the samples
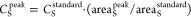
1Absolute protein quantification by ICP-MS requires maintaining the elemental response factor constant along the complete chromatographic analysis. To fulfill this requirement, a total consumption nebulizer (capillary LC interface, Agilent) was used between the capHPLC and ICP-MS/MS systems.^71^ For signal variation correction (<6% relative standard deviation) and enhanced sensitivity,^72^ continuous addition of 50 mL/min carbon dioxide (CO_2_/Ar, 10:90) gas mixture (Air liquide, Madrid, Spain) to the ICP-MS plasma was controlled with a Bronkhorst Mass Flow Meter (the Netherlands). CO_2_/Ar was mixed on-line with optional gas O_2_/Ar (20:80) (Air liquide, Madrid, Spain) through a T-connection located between the exit of the ICP-MS optional gas and the optional inlet of the nebulization chamber.

#### 3.4.1 Correlation between the Sulfur and the Protein Chromatographic Profiles

Knowledge of the stoichiometry of sulfur atoms in a protein sequence is needed to transform sulfur concentration into protein concentration. For this purpose, the identity of the toxins eluted along the chromatographic separation of venom was achieved through parallel ESI-MS native mass profiling in the same chromatographic peaks analyzed by ICP-MS/MS. ESI-MS mass profiling was recorded with a Bruker Daltonics (Bremen, Germany) ESI-QToF MS Impact II instrument. Protein identification was inferred through a comparison of the masses of *Walterinnesia* venom proteins assigned by bottom-up and top-down proteomics analyses (Supporting Information Tables S2–S5) with those gathered through venom gland transcriptomic-assisted top-down analysis of a *W. aegyptia* venom sample (Supporting Information Table S1) deposited in NCBI SRA and TSA databases associated with BioProject PRJA506018. LC–MS/MS.raw and centroid.mzXML data are publicly available in the MassIVE repository under the accession number MSV000081885 (ftp://massive.ucsd.edu/MSV000081885) and ProteomeXchange (accession number PXD008597).^73^

## 4 Results and Discussion

### 4.1 Experimental Design

**Figure 1:**
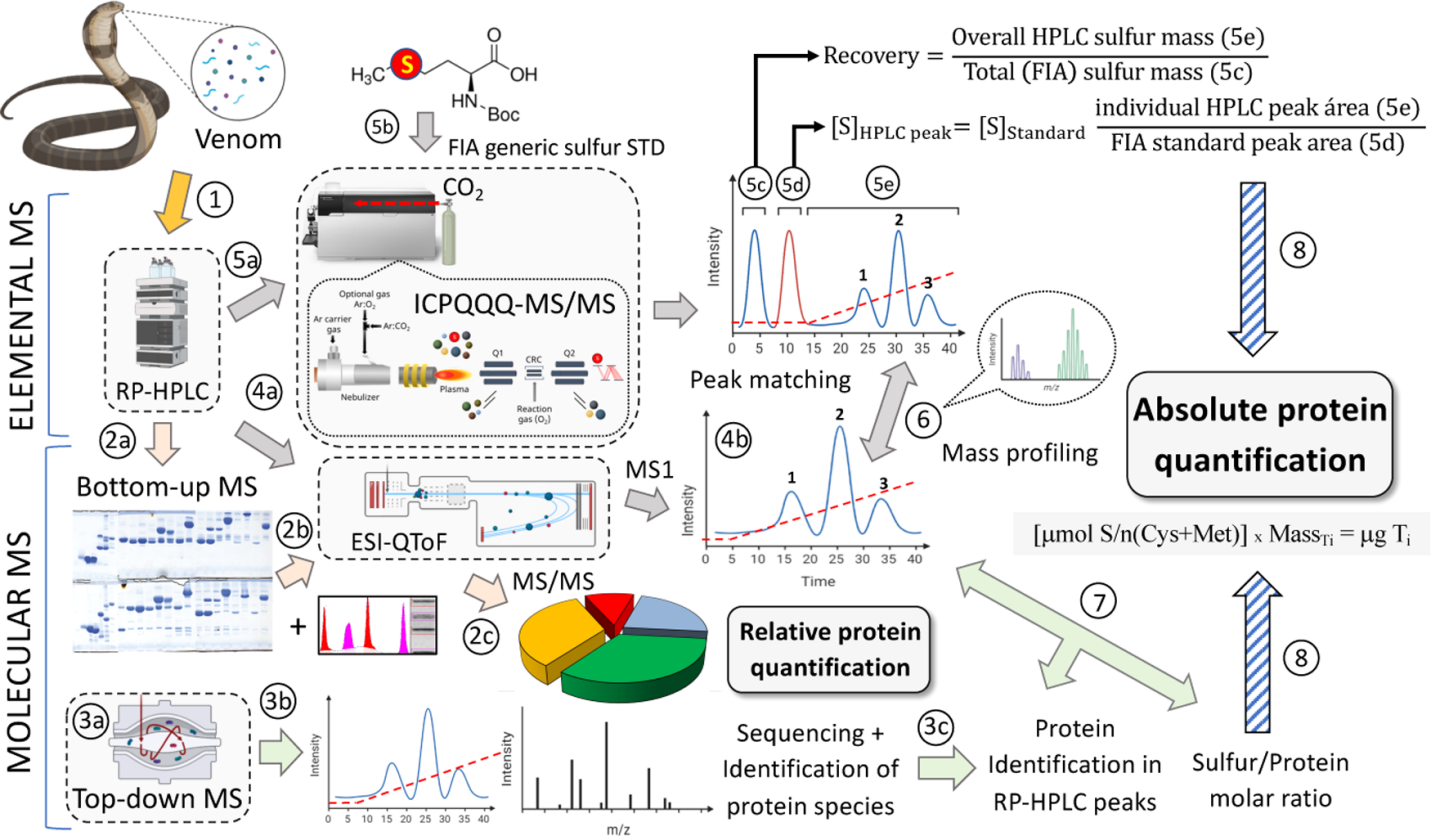
Schematic of the combined molecular and elemental mass spectrometry methodology applied in this work for the absolute quantification of the venom proteomes of black desert cobras, *W. aegyptia* and *W. morgani*. The workflow comprises four RP-HPLC venom protein separations and downstream analysis through bottom-up (2a–c) and top-down (3a–c) venomics and combined parallel mass profiling (4a,b and 6) and absolute sulfur determination by ICPQQQ-MS/MS (5a–e). Continuous sulfur quantification along the chromatographic run was correlated with the molecular masses measured in the parallel RP-capHPLC-ESI-QToF run for the parent venom toxins (6) and assigned to amino acid sequences gathered from bottom-up and top-down venomics (7). Molar ratios sulfur/protein [μmol S/*n*(Cys + Met)] computed throughout the chromatogram were translated into the corresponding absolute protein amounts (8) using the equation [μmol S/*n*(Cys + Met)] × *M*_Ti_ = μg Ti, where *n*(Cys + Met) is the number (*n*) of cysteine and methionine residues in the amino acid sequence of toxin “*i*” (*T_i_*) and *M*_Ti_ is the ESI-MS determined monoisotopic molecular mass of toxin *i*.

**Figure 2:**
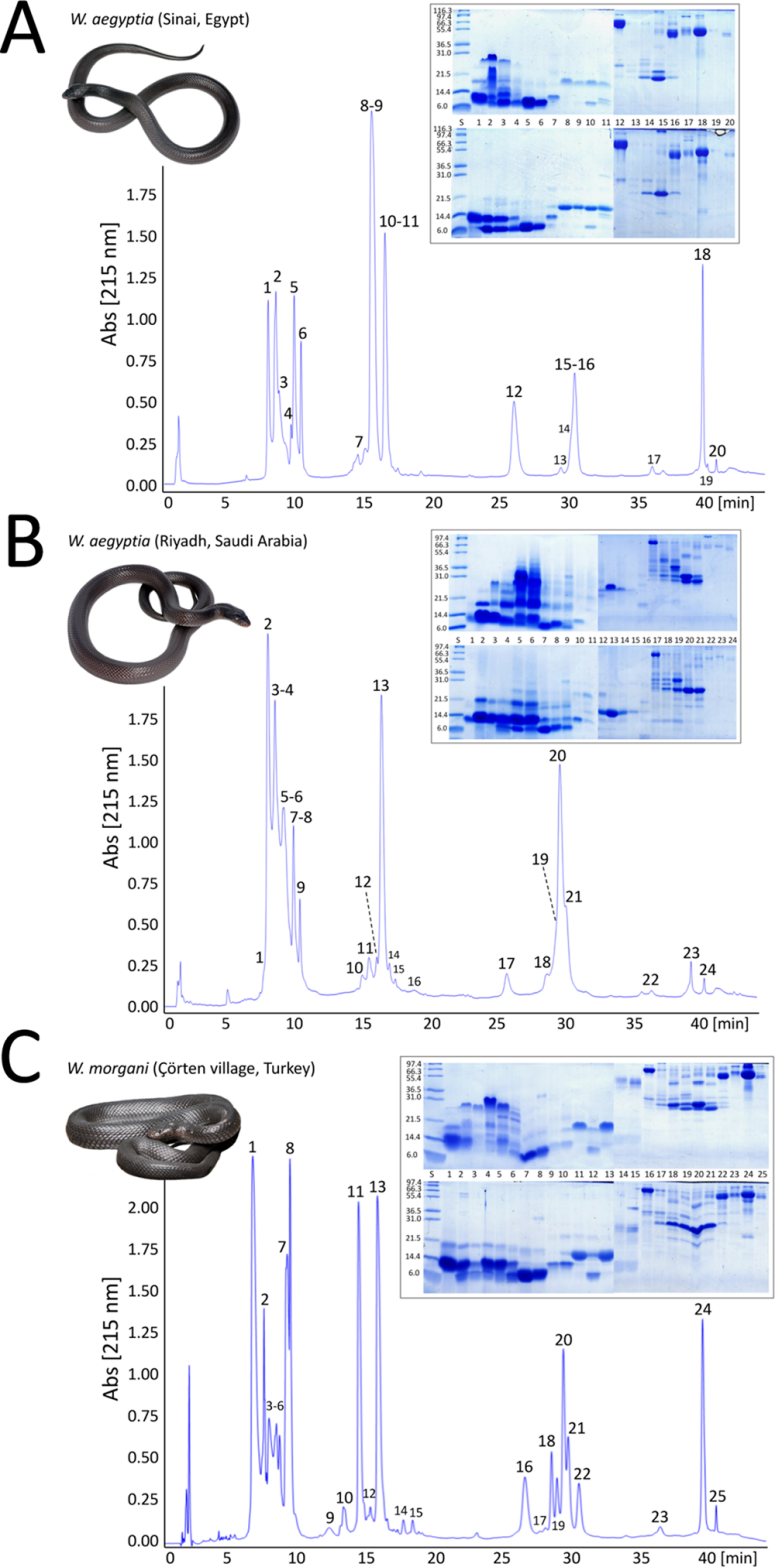
Bottom-up venomics analysis of the toxin arsenal of desert black cobras, *W. aegyptia* and *W. morgani*. Panels (A–C) display, respectively, reverse-phase chromatographic separations of the venom proteins of two *W. aegyptia* specimens (Sinai Peninsula, Egypt, and Riyadh, Saudi Arabia) and a venom sample from a *W. morgani* specimen original from Çörten village (Turkey). For venomics analyses, chromatographic fractions were collected manually and analyzed by SDS-PAGE (inset) under nonreduced (upper panels) and reduced (lower panels) conditions. Protein bands were excised, in-gel digested with trypsin, and the resulting proteolytic peptides were fragmented through LC-*n*ESI-MS/MS. Parent proteins were identified by database searching (against the last update of the NCBI nonredundant database, including the *W. aegyptia* venom gland transcriptomic data deposited with the SRA and TSA databases, Supporting Information Table S1) and *de novo* sequencing followed by BLAST analysis (Supporting Information Tables S2–S4). Picture of *W. aegyptia* specimens displayed in panels (A) and (B) were taken by Salvador Carranza. Picture of *W. morgani*, Bayram Göçmen.

**Figure 3:**
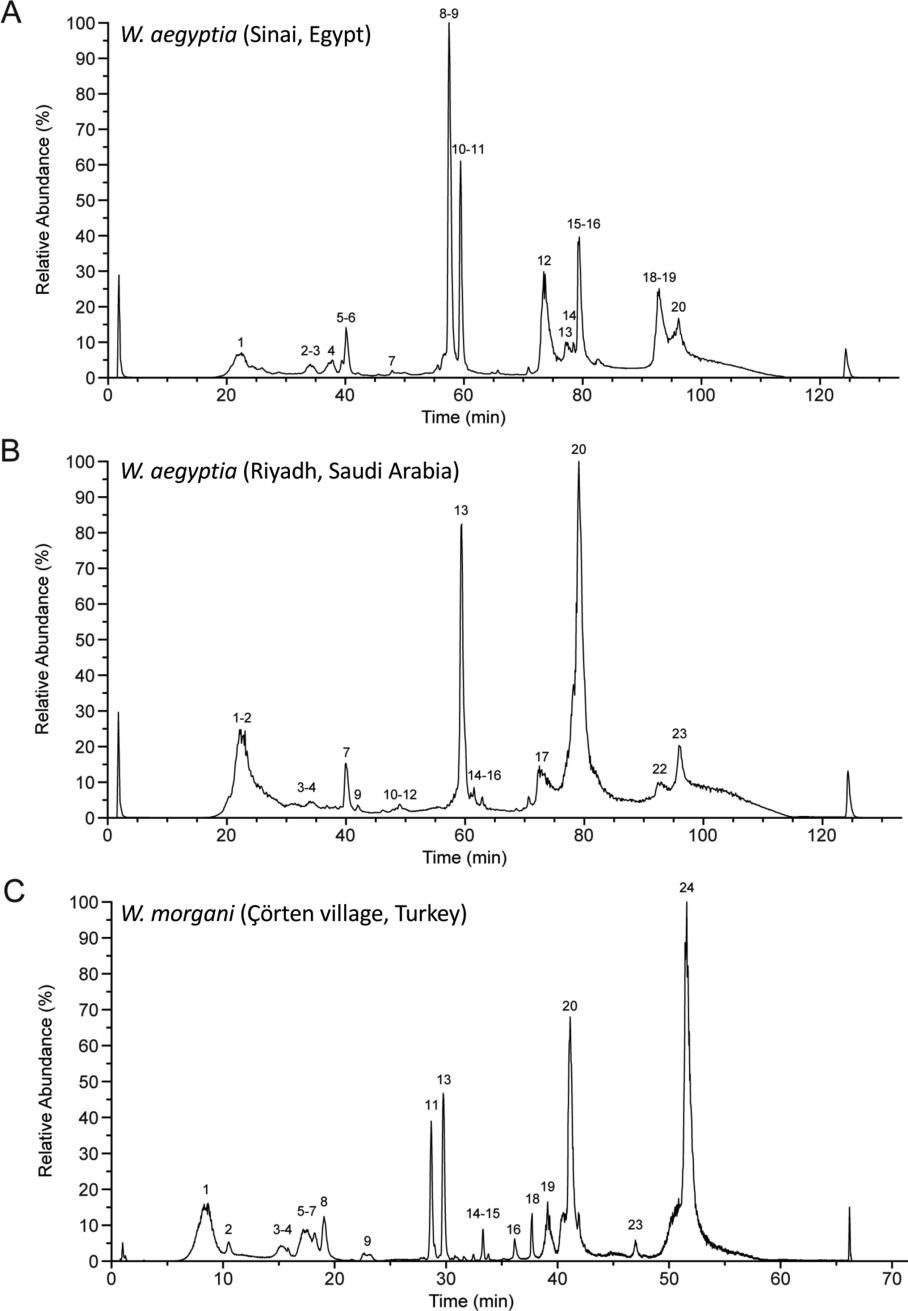
Total ion current (TIC) profiles of reduced venom proteins of Egyptian and Saudi Arabian *W. aegyptia* (panels A and B, respectively) and Turkish *W. morgani* (panel C) separated by reverse-phase HPLC. Peak numbering same as in the homologous UV-monitored chromatographic traces displayed in Figure 2. Top-down MS identifications of proteins in the proteomes of *W. aegyptia* and *W. morgani* venoms are listed in the Supporting Information Table S5 and integrated with the homologous bottom-up datasets in the Supporting Information Tables S2–S4.

We report the application of a novel MS-based workflow for the absolute quantification of the locus-resolved venom proteomes of two species of desert black cobras, *W. aegyptia* and *W. morgani*. The experimental setting, schematized in Figure 1, includes decomplexation of the venom samples by reverse-phase chromatography independently coupled to each of four mass spectrometry systems. Protein identification was accomplished through a combination of bottom-up (Figure 1, 2a–c) and top-down (Figure 1, 3) molecular MS-based workflows. Bottom-up venomics (BUV) relies on in-gel tryptic digestion of SDS-PAGE bands of the venom proteins separated using RP-HPLC (Figure 1, 2a), ESI-MS/MS sequencing of the resulting tryptic peptides (Figure 1, 2b), and matching the recorded product ion spectra against a protein database with a search algorithm.^32,59,63^ BUV takes advantage of venom fractionation to simultaneously quantitate the relative abundances of the different venom components (Figure 1, 2c). On the other hand, in the top-down venomics (TDV) approach (Figure 1, 3a–c) front-end-fractionated disulfide-bond-reduced intact polypeptide ions generated by electrospray ESI are manipulated and dissociated inside a high-resolution Fourier transform ion-trapping (e.g., orbitrap) mass spectrometer (Figure 1, 3a).^64,65,74^ A benefit of TDV is that the intact mass of every proteoform is retained, overcoming the challenge of BUV regarding the characterization of small proteins that often yield an insufficient number of proteolytic peptides for unequivocal proteoform identification. Locus-resolved toxin identification by top-down MS/MS analysis (Figure 1, 3b) complements BUV and represents an important progression toward a full qualitative description of a venom’s proteome (Figure 1, 3c). However, molecular mass spectrometry used in BUV and TDV is inherently not a quantitative technique, and the proposed absolute protein quantification strategies are limited by the need for proteotypic internal standards for each target protein.^32,42,75^ To overcome this limitation, our workflow includes a hybrid elemental and molecular MS configuration where two identical venom samples are submitted to decomplexation through parallel RP-μHPLC run under identical chromatographic conditions. The identity of the toxins along the chromatographic separation was inferred through ESI-QToF mass profiling (Figure 1, 4a) matching the monoisotopic molecular masses calculated for mature toxins recorded in the BUV and TDV analyses or calculated from a homologous venom gland transcriptomic database^73^ (Supporting Information Table S1) (Figure 1, 4b). For the absolute quantification of the venom’s proteins, sulfur concentration was continuously measured throughout the chromatogram via ICP-MS/MS (Figure 1, 5a). Then, the sulfur response factor obtained from a certified S-containing generic compound (Figure 1, 5b) injected using capFIA prior to the capHPLC analysis (i.e., the peak area of S per unit of concentration of the S standard injected) was used to translate the individual peak areas of the different peaks into sulfur concentration (Figure 1, 5d,e). Compared to previous ICP quantification approaches using online isotope dilution analysis (IDA) to keep both the protein response factor and the isotopic tracer added continuously constant along the whole chromatogram, the recently introduced strategy of continuous addition of 50 mL/min carbon dioxide (CO_2_/Ar, 10:90) gas mixture to the plasma provides excellent signal variation corrections along the chromatographic separation for all elements simultaneously (<6 RSD%) while maintaining sensitivity enhancement (2–9-fold).^43,71^ This approach makes the use of isotopic dilution analysis unnecessary, thereby simplifying the mathematical treatment of the data (Figure 1, 5c,d). Sulfur quantified along the chromatographic run was assigned to the parent venom proteins separated in the parallel RP-capHPLC-ESI-QToF run (Figure 1, 6), and the stoichiometry S/P [mol S (Cys + Met)/mol Protein] was computed throughout the chromatogram from the amino acid sequences (Figure 1, 7) and translated into the corresponding absolute protein amounts (Figure 1, 8).

### 4.2 Combined Bottom-Up and Top-Down MS Characterization and Relative Quantification of *W. aegyptia* and *W. morgani* Venom Proteomes

Combined bottom-up and top-down MS approaches were applied to match the RP-HPLC-separated venom profiles of two *W. aegyptia* specimens (Sinai Peninsula, Egypt, and Riyadh, Saudi Arabia) and a venom sample from a *W. morgani* specimen originating from Çörten village (Turkey) to a *W. aegyptia* venom gland transcriptomic database. Figures 2 and 3 display RP-HPLC decomplexation of the venom proteomes of the Egyptian and Saudi Arabian *W. aegyptia* (panels A and B, respectively) and Turkish *W. morgani* (panel C). Figure 2 displays the bottom-up venomics analysis of these three desert black cobras’ venom proteomes applying primary RP-HPLC snake venom protein separations with eluate detection at the peptide bond absorbance wavelength (215 nm) and secondary subfractionation of the chromatographic peaks by SDS-PAGE. Figure 3 displays the total ion current (TIC) profiles of the same venom samples analyzed in Figure 2. The simple comparison of the separations of the same proteomes visualized by quantifying different parameters clearly shows large differences between the relative abundances of the venom components as a function of the monitored parameter, absorbance vs TIC. The reason for this discrepancy has to be attributed to the different physical principles underlying the techniques used to monitor similar RP-HPLC eluates. Monitoring the reverse-phase column eluate at the absorbance wavelength of the peptide bond provides a measure of the concentration of peptide bonds along the chromatographically separated fractions. The relative abundances of a venom toxin arsenal estimated as the ratio of the peak area to the total area of the venom proteins in the reverse-phase chromatogram have a unit of “% of the total chromatographic peptide bond concentration,” which conceptually is a proxy of the weight % “g toxin*_i_*/100 g of total venom proteins.”^41^ On the other hand, the TIC chromatograms recorded through TDV (Figure 3) represent the summed dimensional intensity across the entire range of masses detected at every point of the RP-HPLC chromatogram. Different ionization efficiency/detectability intrinsic to polypeptide ions limit the applicability of TIC to estimate relative protein abundances.^76,77^ Hence, top-down MS data were used here only for the purpose of complementing and expanding the bottom-up qualitative identification of the different proteins/proteoforms present in the three desert black cobra venom proteomes sampled (Supporting Information Tables S1–S5).

**Figure 4:**
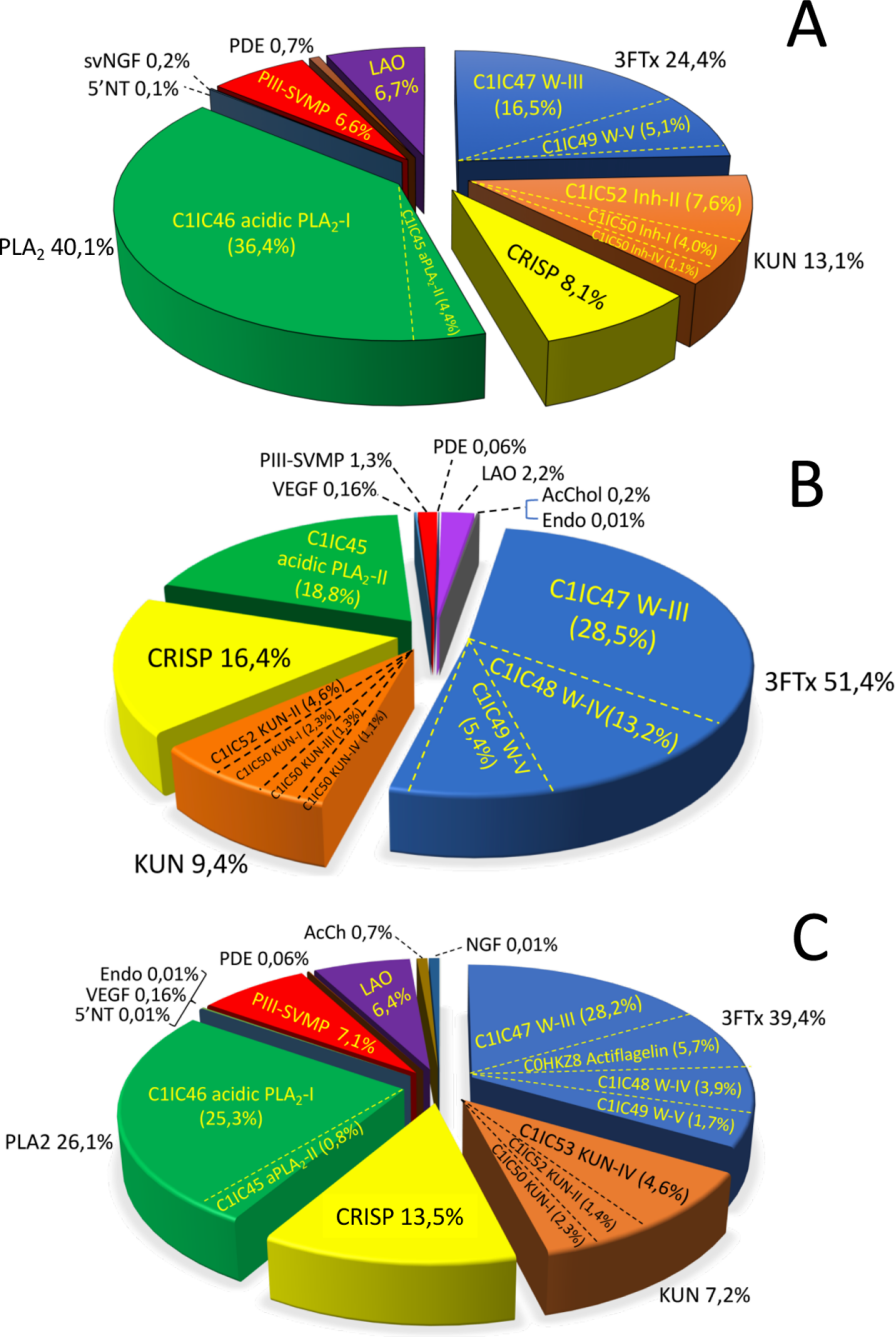
Pie charts displaying the BUV quantified relative occurrence (in the percentage of total venom proteins) of the different protein families in the venom proteome of desert black cobras, *W. aegyptia* (Sinai Peninsula, Egypt) (panel A), *W. aegyptia* (Riyadh, Saudi Arabia) (panel B), and *W. morgani* (Çörten village, Turkey) (panel C). Major TDV-identified family member components are highlighted in each pie chart. Acronyms: 3FTx, three-finger toxin; KUN, Kunitz-type serine proteinase inhibitor-like protein; CRISP, cysteine-rich secretory protein; PLA_2_, phospholipase A_2_; 5′NT, 5′ nucleotidase; svNGF, snake venom nerve growth factor; PIII-SVMP, snake venom metalloproteinase of class PIII; PDE, phosphodiesterase; LAO, l-amino acid oxidase; VEGF, vascular endothelial growth factor; AcChol, acetylcholinesterase; Endo, endonucleotidase.

Figure 4 displays a comparison of the relative abundances of the toxin families comprising the venom proteomes of the *W. aegyptia* and *W. morgani* venom proteomes quantified by BUV, and the identity of the major toxin family members was gathered by TDV (Supporting Information Tables S2–S4). The three venoms are made up of four dominant toxin families, three-finger toxin (3FTx, 24–51%), phospholipase A_2_ (PLA_2_, 19–41%), cysteine-rich secretory protein (CRISP, 8–16%), and Kunitz-type serine proteinase inhibitor-like protein (KUN, 7–13%). Two other toxin families, snake venom metalloproteinases of class PIII (PIII-SVMP) and l-amino acid oxidase (LAO), are present in medium abundances (6–7%) in the venom proteomes of *W. aegyptia* from Sinai Peninsula (Egypt) and *W. morgani*, and the three venoms contain a set of 3–6 low-abundance (<0.1%) proteins, including 5′-nucleotidase (5′NT), endonuclease (Endo), phosphodiesterase (PDE), nerve and vascular endothelial growth factors (NGF and VEGF), and acetylcholinesterase (AcChol) (Figure 4; Supporting Information Tables S2–S5). The small set of individual major toxins that make up the venom protein families, 3FTx (2–4 proteins), KUN (3–4 proteins), and PLA_2_ (two highly homologous molecules), is highly conserved among the three *Walterinnesia* venom proteomes, but their relative abundances vary (Figure 4). Although lethal doses for the individual *Walterinnesia* venom toxins have not been reported, making an informed discussion on the impact of compositional variability on the overall toxicity of the venoms impossible, the i.p. murine LD_50_ of *W. morgani* venom 0.66 (CI_95 %_ 0.13–3.37) μg/g mouse body weight (this work) is comparable to the i.v. LD_50_ reported for *W. aegyptia* (0.79 (0.62–1.09) μg/g mouse).^73^

### 4.3 Absolute Venom Protein Quantification via a Hybrid Elemental and Molecular Mass Spectrometry Configuration

The development during the first decade of the 21st century^33^ of quantitative MS-based strategies represented a major advance in the proteomics arena. An inherent drawback of absolute protein quantification based on molecular MS approaches is the requirement of stable isotope-labeled analogous standards for each target molecule.^37,78^ In this context, since its introduction in the early 2000s,^79^ heteroatom-tagged elemental MS is gaining momentum as a versatile technique for the absolute quantification of biomolecules without specific standards due to its capability to quantify heteroatoms (any element except C, H, N, O, and F) present in the structure of the target biomolecules.^72,80,81^ Leveraging on a new approach for removing polyatomic interference using a triple quadrupole inductively coupled plasma (ICP) mass spectrometry configuration,^69^ we have recently developed a hybrid elemental and molecular MS platform based on a reverse-phase (RP) capillary μHPLC hyphenated to an ICP-MS/MS mass spectrometer and on-line IDA for the absolute quantification of the venom proteomes of the Mozambique spitting cobra (*Naja mossambica*), the black-necked spitting cobra (*Naja nigricollis*), the New Guinea small-eyed snake (*Micropechis ikaheka*), and the Papuan black snake (*Pseudechis papuanus*).^40,41^ In this approach, the combination of spiking a generic S-containing internal standard to the sample and postcolumn addition of ^34^S provided the basis for the absolute quantification of the RP-HPLC-separated S-containing venom toxins, the identity of which was accomplished by ESI-QToF mass profiling along a parallel RP-HPLC run.^32,71,72^ Now, we have applied a newly developed protocol where the addition of 50 mL/min carbon dioxide (CO_2_/Ar, 10:90) gas mixture directly to the plasma abolishes the need for correcting sulfur response factor variation (>6%) along the chromatographic separation using complex ^34^S-isotope dilution procedures.^42,43^

**Figure 5:**
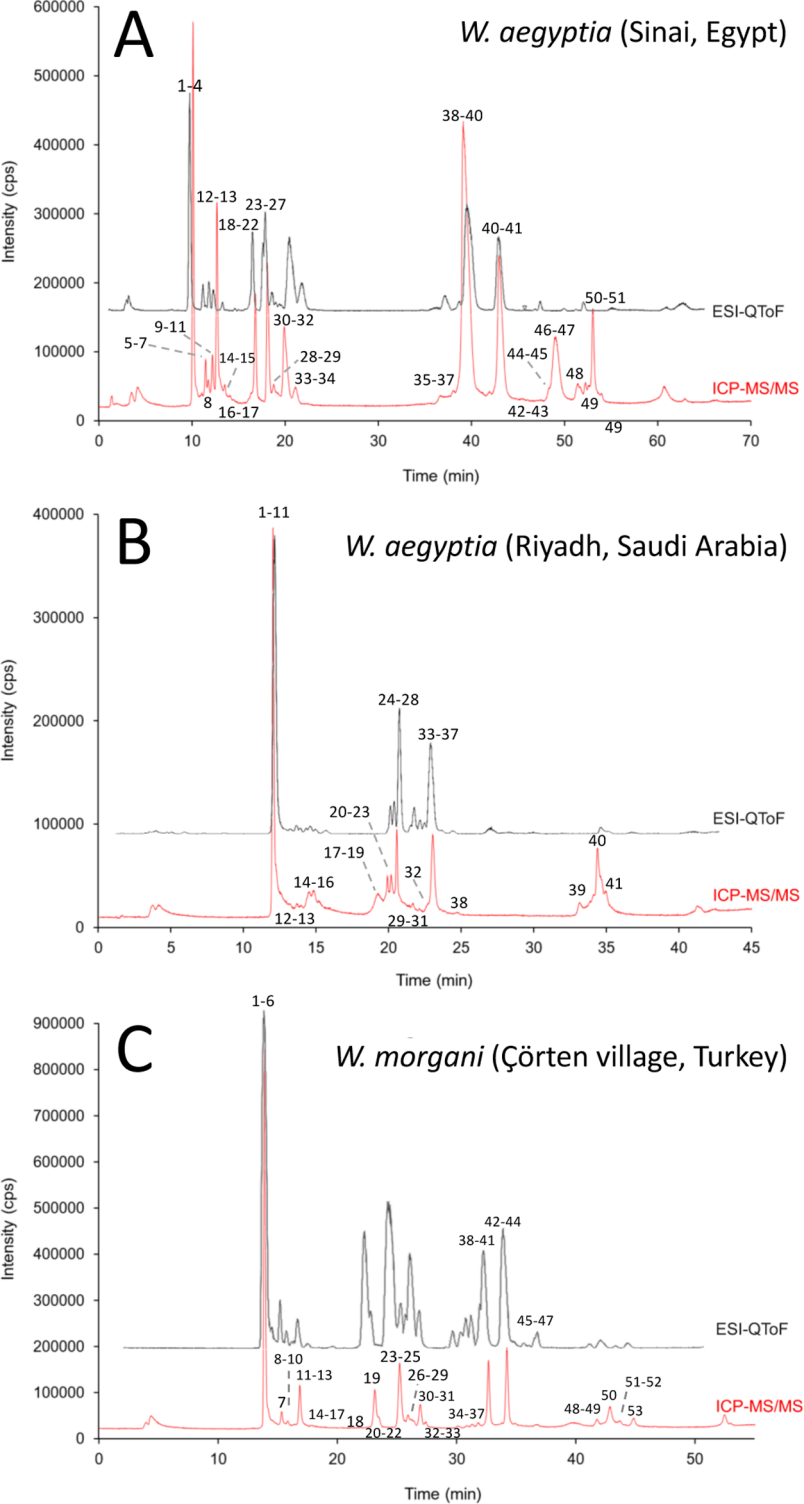
Overlay of the ICP-MS mass flow chromatograms (red) and the ESI-MS chromatograms (black) of the venoms of (A) *W. aegyptia* (Sinai Peninsula, Egypt), (B) *W. aegyptia* (Riyadh, Saudi Arabia), and (C) *W. morgani* (Çörten village, Turkey). Peak matching displayed in the Supporting Information Tables S6–S8 enabled correlating molecular peak identity and elemental S quantitation.

Our current workflow retains the hybrid elemental and molecular MS configuration (Figure 1, 4a and 5a) of its predecessor platform (Figure 1 of Calderón-Celis et al.).^41^ Complete chromatographic column protein recovery is a strictly necessary condition to achieve accurate ICP-MS-based generic absolute protein quantification. Sample recoveries from the C4 capHPLC column, evaluated via Flow Injection Analysis, were, respectively, 95 ± 3, 92 ± 1, and 102 ± 1% for *W. aegyptia* (Sinai Peninsula), *W. aegyptia* (Riyadh), and *W. morgani* (Çörten village). ICP-MS sulfur quantification along the chromatographic run was assigned to the parent venom proteins separated in the parallel RP-capHPLC-ESI-QToF run (Figure 1, step 6) and was then translated into the corresponding absolute protein amounts (e.g., mg, μmoles) (Figure 1, step 8; Supporting Information Tables S6–S8) using the stoichiometry mol S (Cys + Met)/mol Protein computed throughout the chromatogram from the amino acid sequences, gathered from the bottom-up and top-down venomics analyses. Table 1 compares the absolute ICP-MS/MS quantifications of the major toxins of *W. aegyptia* (Sinai Peninsula), *W. aegyptia* (Riyadh), and *W. morgani* (Çörten village) venoms, expressed as mg toxin/100 mg venom, with the respective relative quantifications gathered through our three-step bottom-up venom proteome quantification protocol (Materials and Methods Section 3.3.1).^60,63^ The reasonably good agreement between the values obtained by ICP-MS/MS and bottom-up venomics for major toxin families (Table 1) corroborated our previous assumption^41^ that the “% of the total venom proteome’s peptide bonds” represents a proxy for the weight % (g/100 g) for the venom components and thus has units of mg toxin/toxin family per 100 mg of total venom proteins. Of course, this strategy is still prone to error, given that the contribution to the molar absorption coefficient (ε) of each protein species is not solely determined by its peptide bonds, but the contribution of several amino acid residues must be taken into account as well.^82^ This miscalculation does not occur in ICP quantification because the signal is directly proportional to the concentration of sulfur. The relative abundances calculated from the mass signal intensity (cps, counts per second, the number of ions that hit the detector per unit of time) recorded in the ESI-QToF mass profiling analysis (Figure 1, step 6) did not show a consistent correlation with the ICP-MS/MS data (Table 1). No toxin/family of toxins-associated pattern emerges from the data displayed in Table 1 that would allow rationalizing and thus eventually correcting the observed biases. On the other hand, for all pairwise comparisons of homologous data obtained by ICP-MS and by our bottom-up snake venomics approach,^32,60,63^ the standard deviation of the averaged value was within the range of 0.1–3.3% (Table 1).

**Table 1:** Relative Quantification through Bottom-Up Venomics and Absolute Quantification via ICP-MS/MS of the Major and Some Minor Components of the Venom Proteomes of the Desert Black Cobras, *W. aegyptia* (Sinai Peninsula, Egypt), *W. aegyptia* (Riyadh, Saudi Arabia), and *W. morgani* (Çörten village, Turkey)^a^

		ICP-MS/MS [mg/100 mg V]	BUV [% venom proteome]	mean ± SD	ESI-QToF [% cps]
*W.aegyptia* (Sinai)	3FTX	22.6	24.4	23.5 ± 0.9	65.8
	KUN	13.8	13.1	13.5 ± 0.4	28.5
	PLA_2_	40.6	40.1	40.3 ± 0.3	3.8
	CRISP	9.8	8.1	8.9 ± 0.8	0.6
	svNGF	0.5	0.2	0.3 ± 0.1	0.2
	PIII-SVMP	12.6	6.6	9.6 ± 2.9	1.1
	LAO		6.7		
	5′NT		0.1		
	PDE		0.7		
*W. aegyptia* (Riyadh)					
	3FTX	54.8	51.4	53.1 ± 1.7	61.0
	KUN	16.0	9.4	12.7 ± 3.3	23.1
	PLA_2_	12.6	18.8	15.7 ± 3.1	14.9
	CRISP	16.0	16.4	16.5 ± 0.1	1.0
	PIII-SVMP		1.3		
	LAO		2.23		
	VEGF		0.16		
	PDE		0.06		
	AcCHOL		0.2		
	Endo		0.01		
*W. morgani*					
	3FTX	45.4	39.4	42.4 ± 3.0	65.4
	KUN	13.7	7.2	10.5 ± 3.2	10.7
	PLA_2_	27.0	26.1	26.6 ± 0.5	21.4
	CRISP	13.8	13.5	13.7 ± 0.2	1.9
	svNGF	0.001	0.010	0.0056 ± 0.0044	0.7
	PIII-SVMP		7.1		
	LAO		6.4		
	Endo		0.01		
	VEGF		0.16		
	5′NT		0.01		
	PDE		0.06		
	AcCh		0.7		

a For comparison, the relative abundances calculated from the mass signal intensity recorded in the ESI-QTof mass profiling analysis (cps, counts per second) is also included.

## 5 Concluding Remarks and Perspectives

Established in the 1990s as a powerful analytical technique, molecular mass spectrometry has opened new experimental approaches to address biological questions. However, molecular mass spectrometry is not inherently quantitative, and this analytical deficiency motivated the development of label-free and isotopic labeling methods to determine the relative and absolute abundance of biomolecules in complex biological samples. ICP-MS, a type of elemental mass spectrometry introduced in 1980^83^ and available commercially soon after 1983, is a powerful analytical tool for trace elemental speciation analysis of metals, semimetals, and several nonmetals (and their different isotopes) at concentrations as low as ppq, one part per quadrillion (10^15^).^69,84^ More recently,^36^ ICP-MS has emerged as an alternative to overcome the absolute quantification limitations of molecular MS. Implementation of ICP-MS in the proteomics arena has been delayed by the fact that this technique atomizes the sample and detects individual ionized atomic elements. Therefore, the “elemental” information yielded by ICP-MS cannot *per se* be used to differentiate the different S-donor molecules of a mixture. Notwithstanding its lack of molecular resolution, the omnipresence of sulfur in proteins, together with the fact that proteins can be more and more extensively and efficiently separated nowadays, i.e., by advanced RP-HPLC, make the absolute protein quantification via sulfur determination by ICP-MS a feasible strategy. A major advantage of this approach over molecular MS-based peptide- and protein-centric workflows is that only one generic sulfur-containing standard is sufficient to quantify all of the proteins of a proteome provided the components are sufficiently separated and their amino acid sequences are known. The trend toward hybrid mass analyzer configurations has dominated recent advances in instrumentation. Current hybrid molecular mass spectrometry systems combine the complementary performances offered by in-space beam-type and in-time ion-trapping spectrometers into one instrument.^85^ However, there are no hybrid elemental and molecular mass spectrometry configurations on the market. In this work, we report a novel hybrid instrumental setup to quantify the venom proteomes of the two species of the genus *Walterinnesia*. Along with previous work on the absolute quantification of other snake venom proteomes, it highlights the feasibility of incorporating ICP-MS into hybrid workflows that combine the unique performance of molecular and elemental mass spectrometry, e.g., the unparalleled molecular resolution of top-down MS and the absolute quantification of ICP-MS. Our present work also validates our long-standing strategy for the relative quantification of snake venom proteomes (snake venomics), which primarily estimates the relative abundances of the chromatographically separated fractions as % by weight of the venom toxins/toxin families.^32^

Analytical technological advances have continuously enhanced research on venoms. We would like to think that the analytical advances discussed here toward absolute quantification of snake venom proteomes of moderate complexity may serve as a proof-of-concept for a broader and more routine application of hybrid elemental/molecular MS setups in other areas of the proteomics field Figure 5.

## Supporting Information Available

Transcriptomics database (Table S1); MS/MS identification of peptides/proteins in the RP-HPLC fractions of the venom of adult *W. aegyptia* (Sinai Peninsula, Egypt) (Table S2); MS/MS identification of peptides/proteins in the RP-HPLC fractions of the venom of adult *W. aegyptia* (Riyadh, Saudi Arabia) (Table S3); MS/MS identification of peptides/proteins in the RP-HPLC fractions of the venom of adult *W. morgani* (Çörten village, Turkey) (Table S4); top-down MS identifications of proteins in the proteomes of *W. aegyptia* and *W. morgani* venoms (Table S5); quantification by QQQ ICP-MS of the venom proteome of *W. aegyptia* (Sinai Peninsula, Egypt) (Table S6); quantification by QQQ ICP-MS of the venom proteome of *W. aegyptia* (Riyadh, Saudi Arabia) (Table S7); quantification by QQQ ICP-MS of the venom proteome of *W. morgani* (Çörten village, Turkey) (Table S8) (XLSX)

## Notes

The bottom-up mass spectrometry proteomics data have been deposited to the ProteomeXchange Consortium via the PRIDE^86^ partner repository with the dataset identifiers PXD027495 (Venomics of the desert black cobra *W. aegyptia* from Sinai Peninsula, Egypt), PXD027498 (Venomics of the desert black cobra *W. aegyptia* from Riyadh, Saudi Arabia), and PXD027497 (Venomics of the desert black cobra *W. morgani* from Çörten village, Turkey). Top-down LC–MS/MS.raw and centroid.mzXML data are publicly available in the MassIVE repository under the accession number MSV000086709. LC–MS/MS data can be directly visualized through the GNPS LC–MS dashboard (https://www.biorxiv.org/content/10.1101/2021.04.05.438475v2) and the LC–MS dataset explorer: https://gnps-dataset-explorer.herokuapp.com/ with the Massive identifier MSV000086709 and under the following link: https://gnps-lcms.ucsd.edu/?xicmz=980.8817%3B792.24755859375&xic_formula=&xic_peptide=&xic_tolerance=0.5&xic_ppm_tolerance=10&xic_tolerance_unit=Da&xic_rt_window=&xic_norm=False&xic_file_grouping=GROUP&xic_integration_type=AUC&show_ms2_markers=True&ms2_identifier=MS2%3A1729&show_lcms_2nd_map=True&map_plot_zoom=%7B%7D&polarity_filtering=None&polarity_filtering2=None&tic_option=TIC&overlay_usi=None&overlay_mz=row+m%2Fz&overlay_rt=row+retention+time&overlay_color=&overlay_size=&overlay_hover=&overlay_filter_column=&overlay_filter_value=&feature_finding_type=Off&feature_finding_ppm=10&feature_finding_noise=10000&feature_finding_min_peak_rt=0.05&feature_finding_max_peak_rt=1.5&feature_finding_rt_tolerance=0.3#{%22usi%22:%20%22mzspec:MSV000086709:peak/27_Walterinnesia_egyptia_Liverpool_unkown_red_2.mzXML\nmzspec:MSV000086709:peak/27_Walterinnesia_egyptia_Liverpool_unkown_red_1.mzXML\n%22,%20%22usi2%22:%20%22mzspec:MSV000086709:peak/30_Walterinnesia_morgani_Ayse_Turkey_red_1.mzXML\nmzspec:MSV000086709:peak/30_Walterinnesia_morgani_Ayse_Turkey_red_2.mzXML%22}.
